# Potentiation of Antidepressant Effects of Agomelatine and Bupropion by Hesperidin in Mice

**DOI:** 10.1155/2018/9828639

**Published:** 2018-10-28

**Authors:** Jegan Sakthivel Nadar, Pravin Popatrao Kale, Pramod Kerunath Kadu, Kedar Prabhavalkar, Ruchita Dhangar

**Affiliations:** ^1^Department of Pharmacology, SVKM'S Dr. Bhanuben Nanavati College of Pharmacy, V. M. Road, Mithibai Campus, Vile Parle West, Mumbai 400 056, India; ^2^Department of Pharmaceutics, SVKM'S Dr. Bhanuben Nanavati College of Pharmacy, V. M. Road, Mithibai Campus, Vile Parle West, Mumbai 400 056, India

## Abstract

Hesperidin, a well-known flavanone glycoside mostly found in citrus fruits, showed neuroprotective and antidepressant activity. Agomelatine, a melatonergic MT_1_/MT_2_ agonist and 5-HT_2C_ receptor antagonist, exhibits good antidepressant efficacy. Bupropion has been widely used for the treatment of depression because of its dopamine and norepinephrine reuptake inhibition. The objective of present study was to assess the antidepressant effects of hesperidin combination with agomelatine or bupropion. Male Swiss Albino mice received treatment of saline, vehicle, ‘hesperidin alone', ‘agomelatine alone', hesperidin+agomelatine, ‘bupropion alone', hesperidin+bupropion, and agomelatine+bupropion for 14 days. The immobility period was analysed 30 min after the treatment in forced swim and tail suspension tests. Dopamine and serotonin levels were analysed in hippocampus, cerebral cortex, and whole brain using HPLC with fluorescence detector. Hesperidin plus agomelatine treated group was better in terms of decrease in immobility period and increase in dopamine and serotonin levels when compared to their respective monotherapy treated groups.

## 1. Introduction

Depression is a common mental disorder that affects thought, behaviour, feeling, and physical well-being of an individual. It is projected that by 2030 depression will be the foremost contributor among the worldwide burden of diseases [1]. Despite introduction of several novel classes of antidepressant drugs, advances in understanding the psychopharmacology, and biomarkers of major depression, only 60%–70% of patients with depression respond to antidepressant therapy. Of those who do not respond, 10%–30% exhibit treatment-resistant symptoms with difficulties in occupational and social function, suicidal thought, decline of physical health, and increased health care utilization [2]. Though they have different mechanisms of action, all present antidepressants ultimately produce the same final rates of response and remission [3]. Severe and intolerable side effects of available antidepressants and limited success rate (60–70%) of first-line monotherapy drugs have resulted in preference of potentiation or augmentation therapy in treatment for depression [4].

Hesperidin, a naturally occurring flavanone glycoside, is mainly found in citrus fruits [5]. Therapeutically useful properties of hesperidin have been described as antioxidant [6, 7], neuroprotective [8], antineoplastic [9], and anti-inflammatory [10]. Hesperidin interacts with the serotonergic 5-HT_1A_ receptor and elevates serotonin level thereby producing antidepressant effect [11]. Also hesperidin has been found to increase hippocampal BDNF levels, suggesting a possible involvement of neurogenesis [12]. As per latest findings, the antidepressant effect of hesperidin is also dependent on NO/cGMP pathway [12]. Hesperidin at 1mg/kg or lower doses significantly reduced immobility time in forced swim test in mice [13].

Agomelatine is another antidepressant agent with a novel mechanism of action. It acts as a potent agonist at the melatonergic receptors MT_1_, MT_2_ [14] and as an antagonist at the 5-HT_2C_ receptor [15]. Agomelatine acts on melatonin receptors in the suprachiasmatic nucleus and normalizes circadian rhythms, thereby improving sleep and resynchronizing disrupted circadian rhythms [16]. It enhances basal prefrontocortical dopaminergic and noradrenergic transmission as well as increasing basal noradrenaline release in the dorsal hippocampus by 5-HT_2C_ receptor antagonism [15]. It also decreases stress-induced glutamate release in the prefrontal cortex (PFC) and increases brain-derived neurotrophic factor (BDNF) in hippocampus and PFC [17, 18]. Agomelatine is an effective antidepressant with a rapid onset of action not only in patients with Major Depressive Disorder (MDD) but also in patients with severe MDD, seasonal affective disorder, bipolar I disorder, and generalized anxiety disorder [19]. Compared to other antidepressants, agomelatine does not cause sexual dysfunction or worsening of sleep disturbances and has fewer side effects [20]. Agomelatine treatment in forced swim test resulted in significant reduction in the immobility period at 8 mg/kg dose [21].

Bupropion is a preferential norepinephrine and dopamine reuptake inhibitor [22]. It is also an antagonist to neuronal nicotinic acetylcholine receptors [23]. Bupropion is considered within the 5 most prescribed antidepressants [24]. It has better efficacy, safety, and tolerability with fewer side effects when compared to antidepressant-like fluoxetine, paroxetine, and venlafaxine. Bupropion showed significant decrease in immobility period at 10 mg/kg dose [25, 26]. Therefore, the objective of present study was to assess the effect of hesperidin combination with agomelatine or bupropion in the treatment of depression.

## 2. Materials and Methods

### 2.1. Animals

Male Swiss Albino mice weighing 22–27 gm were procured from Bharat Serums and Vaccines Pvt. Ltd. Thane. They were housed in groups of 6 in polycarbonate cages at SVKM's Animal Facility (12 h: 12 h light/dark cycle, room temperature 20-22°C, and humidity 75 ± 5 %). They had free access to standard food and water. Animals were allowed to adapt themselves to the new environment for one week prior to the start of the experimental works. Experiments were approved by Institutional review committee for use of animal subjects (Approval number- CPCSEA/IAEC/BNCP/P-19/2014).

### 2.2. Drug Solution and Treatments

Agomelatine (Watson Pharmaceuticals Pvt. Ltd.) was homogeneously suspended in a 1 % solution of hydroxyethyl cellulose and bupropion (Aurobindo Pharma Pvt. Ltd.) was dissolved in normal saline (0.9 % W/V NaCl). Hesperidin (Otto Chemie Pvt. Ltd.) was dissolved by the sequential addition of dimethyl sulfoxide (DMSO) up to a final concentration of 5 %, a water solution of 0.25 % Tween 80 up to a final concentration of 20 %, and saline to complete 100 % volume. Drug solutions were prepared just before each injection session. Vehicle (5 % DMSO + 0.25 % Tween 80 + saline) and drugs were administered intraperitoneally (i.p.) in volumes of 10 ml/kg for 14 days. Each drug was injected separately. Experiments were performed between 11.00 and 17.00 h.

The separate set of animals was used in each experimental model. In each set, mice were randomly divided into 8 groups (n=6 animals/group). Groups I, II, III, IV, V, VI, VII, and VIII received treatment of saline (control group), vehicle (5 % DMSO + 0.25 % Tween 80 + saline), hesperidin (1 mg/kg), agomelatine (8 mg/kg), hesperidin (0.5 mg/kg) + agomelatine (4 mg/kg), bupropion (10 mg/kg), hesperidin (0.5 mg/kg) + bupropion (5 mg/kg), and agomelatine (4 mg/kg) + bupropion (5 mg/kg), respectively. There were 48 mice used for each experimental animal model. The total number of mice was 144 used in present study.

### 2.3. Antidepressant Models

#### 2.3.1. Forced Swim Test

Forced swim test was performed as described by Porsolt et al. [27] on 14th day of treatment schedule. In brief, mice underwent “pretest session” 1 day prior to main tests. They were individually forced to swim for 15 min in a Plexiglas cylinder (10 cm diameter × 25 cm height) with water (22–24°C) at a depth of 18 cm. After 24 hrs, mice were allowed to swim for 6 min and video recorded. Last 5 min session from total 6 min recorded video was used for evaluation. Immobility refers to the cessation of struggling and remaining motionless in the water, making small movements needed to keep the animal's head above the water. After the test, all animals were dried and placed back to the home cage [27].

#### 2.3.2. Tail Suspension Test

The test was performed on 14th day, 30min after administration of drug. Mice were suspended 58 cm above the floor by adhesive tape placed approximately 1 cm from the tip of the tail. The duration of immobility was recorded for a period of 5 min. Mice was considered immobile when they hang passively and completely motionless [28, 29].

### 2.4. Estimation of Dopamine and Serotonin by HPLC with Fluorescence Detector (HPLC-FD) Method

Analysis of dopamine and serotonin levels in brain parts such as cerebral cortex, hippocampus, and whole brain (whole brain = hippocampus + cerebral cortex + remaining brain tissue) was performed using high performance liquid chromatography (Shimadzu, LC-2010C HT, Auto Sampler) with FD (RF-20A-prominence, Shimadzu) method. After 1 hr of treatment, mice were euthanized and heads were dropped in ice cold 0.1 M perchloric acid. Brains were removed and weighed. After separating cerebral cortex, hippocampus, and the remaining brain tissue, they were weighed individually. These were separately homogenized in 2 ml of ice cold 0.1 M perchloric acid. Resulting mixture was centrifuged at 16356 x g (Eppendorf 5810 R, Rotor F-45-30-11) for 30 min (4°C). The obtained supernatant was filtered using 0.45 *μ*m syringe filter and stored at−80°C until the time of analysis. After sample injection, the chromatographic separations were achieved on reversed-phase analytical column (INERTSIL, C18, 5 *μ*m, 25 cm × 0.46 *μ*m) at room temperature. The software LC Solution^@^ was used to process acquired data. The mobile phase was prepared using 0.36 g of potassium dihydrogen orthophosphate and 0.5 ml of phosphoric acid dissolved in 1 litre of millipore water, sonicated, and filtered using a 0.45 *μ*m membrane. Flow rate of mobile phase was optimized at 1.3 ml/min. Dopamine and serotonin levels were detected at an excitation wavelength of 280 nm and an emission wavelength of 315 nm. Dopamine and serotonin peaks were identified by comparing the retention time of sample and standard. The concentrations of dopamine and serotonin in the sample were analysed according to their area under curve and using respective straight line equation. The linearity for dopamine and serotonin was in the range of 0.98–0.997. The unit used to express results was *μ*g/g of wet weight of tissue [26, 30, 31].

### 2.5. Statistical Analysis

The statistical evaluation was performed using the Graphpad InStat for 32 bit Windows version. Groups were compared to assess the statistical significance using one way analysis of variance (ANOVA) followed by Tukey's honest significant difference (HSD) post hoc test in forced swim test and tail suspension test, separately. Two-way ANOVA with Bonferroni was performed using GrphPad Prism 5 for dopamine and serotonin levels in different brain regions. Comparison of all treated was performed against saline treated control group. In addition, comparisons of combination treated groups were done against respective monotherapies. The data is represented as mean ± SEM values and n = 6 per group.

## 3. Results

### 3.1. Forced Swim Test

**Figure 1:**
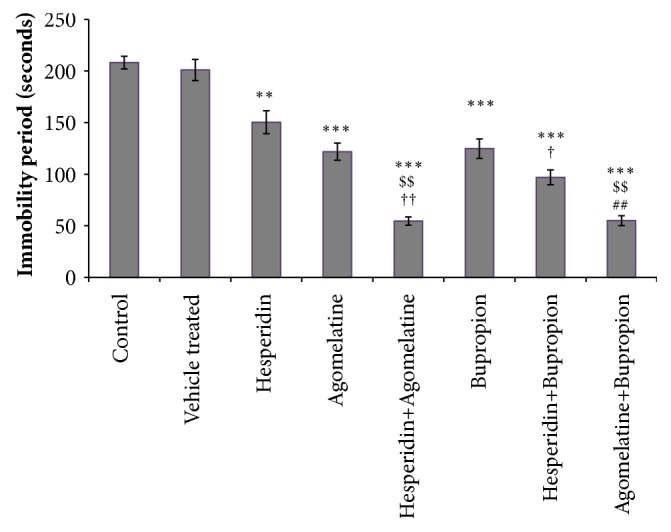
Forced swim test. Significant difference is denoted by *∗∗∗* P<0.001 and *∗∗* P<0.01 as compared to control group; $$ P<0.01 as compared to agomelatine treated group; †† P<0.01 and † P<0.05 as compared to hesperidin treated group; ## P<0.01 as compared to bupropion treated group.

The difference in the immobility period of control and vehicle treated group was not significant. All drug treated groups showed significant decrease in immobility period as compared to control group (Figure 1). Combination treated group hesperidin (0.5 mg/kg) + bupropion (5 mg/kg) showed significant decrease in immobility period, as compared to ‘hesperidin alone' (1 mg/kg) and ‘bupropion alone' (10 mg/kg) treated groups (Figure 1). The immobility period was significantly decreased in hesperidin (0.5 mg/kg) + agomelatine (4 mg/kg) treated group when compared with ‘hesperidin alone' (1 mg/kg) and ‘agomelatine alone' (8 mg/kg) treated groups (Figure 1). Combination of agomelatine (4 mg/kg) + bupropion (5 mg/kg) treated group showed significant decrease in immobility period, as compared to ‘agomelatine alone' (8 mg/kg) treated group (Figure 1).

### 3.2. Tail Suspension Test

**Figure 2:**
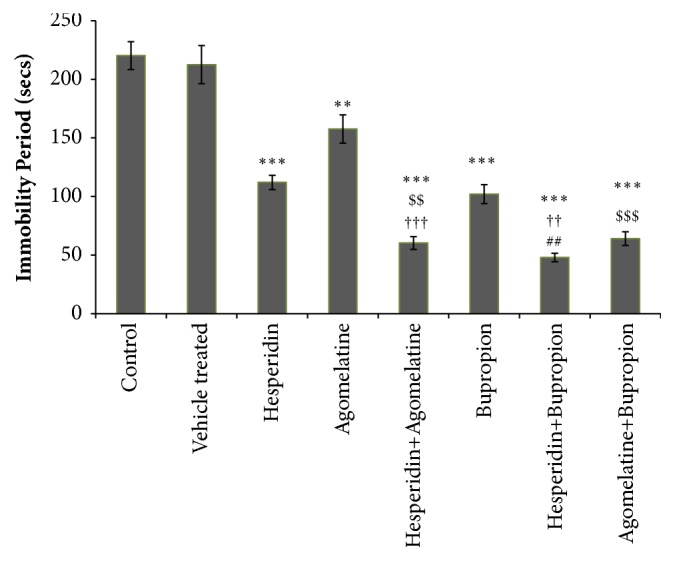
Tail suspension test. Significant difference is denoted by *∗∗∗* P<0.001 and *∗∗* P<0.01 as compared to control group; $$$ P<0.01 and $$ P<0.01 as compared to agomelatine treated group; ††† P<0.001 and †† P<0.01 as compared to hesperidin treated group; ## P<0.01 as compared to bupropion treated group.

The difference in the immobility period of control and vehicle treated group was not significant. The immobility period was significantly decreased in all drug treated groups when compared with control group (Figure 2). Combination treated group hesperidin (0.5 mg/kg) + bupropion (5 mg/kg) showed significant decrease in immobility period, as compared to ‘hesperidin alone' (1 mg/kg) and bupropion alone' (10 mg/kg) treated groups (Figure 2). Combination of hesperidin (0.5 mg/kg) + agomelatine (4 mg/kg) treated group showed significant decrease in immobility period, as compared to ‘agomelatine alone' (8 mg/kg) and ‘hesperidin alone' (1 mg/kg) treated groups (Figure 2). Combination of agomelatine (4 mg/kg) + bupropion (5 mg/kg) treated group showed significant decrease in immobility period, as compared to ‘agomelatine alone' (8 mg/kg) treated group (Figure 2).

### 3.3. Estimation of Dopamine and Serotonin by HPLC with Fluorescence Detector (HPLC-FD) Method

#### 3.3.1. Estimation of Dopamine Level

**Figure 3:**
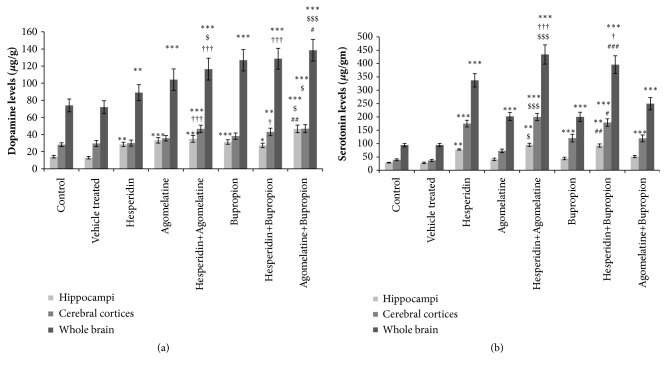
(a) Estimation of dopamine levels in hippocampi, cerebral cortices, and whole brain by HPLC-FD. Significant difference is denoted by *∗∗∗* P<0.001 as compared to control group; $$$ P<0.001 and $$ P<0.01 as compared to agomelatine treated group; ### P<0.001 as compared to bupropion treated group; ††† P<0.001 and †† P<0.01 as compared to hesperidin treated group. (b) Estimation of serotonin levels in hippocampi, cerebral cortices, and whole brain by HPLC-FD. Significant difference is denoted by *∗∗∗* P<0.001 as compared to control group; $$$ P<0.001, $$ P<0.01, and $ P<0.05 as compared to agomelatine treated group; ### P<0.001 and ## P<0.01 as compared to bupropion treated group; †††P<0.001 as compared to hesperidin treated group.

*(1) Hippocampi*. The difference in the dopamine level of control and vehicle treated group was not significant. The dopamine levels were significantly increased in all drug treated groups than control group (Figure 3(a)). Agomelatine (4 mg/kg) + bupropion (5 mg/kg) treatment showed significant increase in dopamine level, as compared to ‘agomelatine alone' (8 mg/kg) and ‘bupropion alone' (10 mg/kg) treated groups, separately (Figure 3(a)).

*(2) Cerebral Cortices*. The difference in the dopamine level of control and vehicle treated group was not significant. The increased level of dopamine in ‘hesperidin alone' (1 mg/kg), ‘agomelatine alone' (8 mg/kg), and ‘bupropion alone' (10 mg/kg) treated groups was not statistically significant than control group. All combination treated groups showed significant increase in dopamine levels, as compared to control group (Figure 3(a)). Dopamine level was significantly increased in hesperidin (0.5 mg/kg) + agomelatine (4 mg/kg) treated group as compared to ‘hesperidin alone' (1 mg/kg) treated group (Figure 3(a)). Combination of hesperidin (0.5 mg/kg) + bupropion (5 mg/kg) treated group showed significant increase in dopamine level, as compared to ‘hesperidin alone' (1 mg/kg) treated group (Figure 3(a)). Agomelatine (4 mg/kg) + bupropion (5 mg/kg) treated group showed significant increase in dopamine level, as compared to ‘agomelatine alone' (8 mg/kg) treated group (Figure 3(a)).

*(3) Whole Brain*. The difference in the dopamine level of control and vehicle treated group was not significant. The dopamine levels were significantly increased in all drug treated groups treated group when compared to control group (Figure 3(a)). Combination treated group hesperidin (0.5 mg/kg) + agomelatine (4 mg/kg) showed significant increase in dopamine level, as compared to hesperidin alone (1 mg/kg) treated group (Figure 3(a)). Combination of hesperidin (0.5 mg/kg) + bupropion (5 mg/kg) showed significant increase in dopamine level, as compared to ‘hesperidin alone' (1 mg/kg) and ‘bupropion alone' (10 mg/kg) treated groups separately (Figure 3(a)). Agomelatine (4 mg/kg) + bupropion (5 mg/kg) treated group showed significant increase in dopamine level, as compared to ‘agomelatine alone' (8 mg/kg) treated group (Figure 3(a)).

#### 3.3.2. Estimation of Serotonin Level

*(1) Hippocampi*. The difference in the serotonin level of control and vehicle treated group was not significant. ‘Hesperidin alone' (1 mg/kg), hesperidin (0.5 mg/kg) + agomelatine (4 mg/kg), and hesperidin (0.5 mg/kg) + bupropion (5 mg/kg) treated groups showed significant increase in serotonin levels, as compared to control group (Figure 3(b)). The serotonin level was significantly increased with hesperidin (0.5 mg/kg) + agomelatine (4 mg/kg) treated group than ‘agomelatine alone' (8 mg/kg) treated group (Figure 3(b)). Combination of hesperidin (0.5 mg/kg) + bupropion (5 mg/kg) treated group showed significant increase in serotonin level, as compared to ‘bupropion alone' (10 mg/kg) treated group (Figure 3(b)).

*(2) Cerebral Cortices*. The difference in the serotonin level of control and vehicle treated group was not significant. All drug treated groups showed statistically significant increase in serotonin levels with the exception of ‘agomelatine alone' (8 mg/kg) treated group, as compared to control group (Figure 3(b)). Combination treated group hesperidin (0.5 mg/kg) + agomelatine (4 mg/kg) showed significant increase in serotonin level, as compared to ‘agomelatine alone' (8 mg/kg) treated group (Figure 3(b)). Hesperidin (0.5 mg/kg) + bupropion (5 mg/kg) treated group showed significant increase in serotonin level, as compared to ‘bupropion alone' (10 mg/kg) treated group (Figure 3(b)).

*(3) Whole Brain*. The difference in the serotonin level of control and vehicle treated group was not significant. All drug treated groups showed significant increase in serotonin levels as compared to control group (Figure 3(b)). Hesperidin (0.5 mg/kg) + agomelatine (4 mg/kg) treated group showed significant increase in serotonin level, as compared to ‘hesperidin alone' (1 mg/kg) and ‘agomelatine alone' (8 mg/kg) treated groups, separately (Figure 3(b)). Serotonin levels were significantly increased in hesperidin (0.5 mg/kg) + bupropion (5 mg/kg) treated group as compared separately to ‘hesperidin alone' (1 mg/kg) and ‘bupropion alone' (10 mg/kg) treated groups (Figure 3(b)).

## 4. Discussion

The results of the present study demonstrate that combination of hesperidin plus bupropion, hesperidin plus agomelatine, and agomelatine plus bupropion showed antidepressant-like effects in both* in vivo* and* in vitro* test models. The* in vivo* tests such as forced swim test and tail suspension test are widely accepted behavioural models for assessing pharmacological antidepressant activity [27, 28]. The antidepressant activity is expressed in terms of immobility period produced due to inescapable condition in forced swim and tail suspension tests, reflecting behavioural despair as seen in human depression [27]. False positive results to psychostimulants, acute drug response, and varying sensitivity for genetic variations are the limitations of these models. These tests are more selective for monoamine-based mechanism analysis and have advantages like being the most predictive and widely used antidepressant models for screening antidepressant activity [27, 32]. The antidepressant effects of hesperidin in combination with agomelatine and bupropion were explored using both the behavioural models. The reduction in immobility period observed in forced swim and tail suspension tests with hesperidin alone, agomelatine alone, and bupropion alone treated groups is in agreement with previous reports [11, 21, 33]. The immobility period was also significantly reduced in all combination treated groups, as compared to control group in both antidepressant models. The reduction in immobility period was better with hesperidin plus agomelatine among combination treated groups in terms of reduction in immobility time period.

As per the Souza et al. [11], hesperidin significantly increased serotonin levels but not dopamine in brain, which may be due to modulation of 5HT-1A receptor. Serotonin levels in present study were significantly increased in hippocampi, cerebral cortices and whole brain which confirm the previous findings. Piacentini et al. [34] reported significant increase in dopamine and serotonin levels after bupropion treatment in the rat hippocampi region. Similar results were observed in the present study. The levels of dopamine were increased in hippocampi and whole brain. Serotonin levels were increased in cerebral cortex and whole brain. The increments observed with bupropion in cerebral cortices for dopamine and hippocampi for serotonin were statistically not significant. These outcomes are in line with previously published in-house study [27]. Reports analysing the effect of agomelatine treatment on dopamine and serotonin in rat prefrontal cortex have shown significant increase in dopamine levels. The present study findings showed increase in dopamine levels; however statistically it was not significant. This might be due to the different experimental conditions or method of estimation than previous reports [15, 35]. The increase in dopamine levels was recorded not only in cerebral cortices but also in hippocampi and whole brain [15, 35]. Thus, agomelatine outcomes are in agreement with the published findings [15, 35]. Combination treated groups showed better serotonin and dopamine profile as compared to respective monotherapy. This may be due to combination of drugs having different mechanisms of action.

Hesperidin metabolism mainly involves CYP450 1A1 and CYP450 1B1 [36]. Other enzymes involved in hesperidin metabolism are CYP450 3A4 and its isoform [37]. About 90% of agomelatine is metabolized by CYP450 1A2 and about 10% by CYP450 2C9 isoform. Bupropion metabolism mainly involves CYP2B6 and to less extent by CYP450 1A2 [38]. Agomelatine may increase the retention of drugs that are metabolized by CYP450 1A2 and CYP450 2C9 and not by CYP1A1 or CYP450 2B6 [35]. Therefore, agomelatine may have low potential for interaction with hesperidin. The interaction between bupropion and hesperidin may also have low potential, as the enzymes involved in metabolism are different. Therefore, the possibility of interaction between hesperidin and agomelatine/bupropion is low.

There is no preclinical or clinical report available with the consideration of combination consisting hesperidin either with agomelatine or bupropion. Suhs et al. [39] have reported beneficial effects of agomelatine and bupropion combination in the treatment-resistant depression. Hesperidin acts through kappa-opioid receptors [13], l-arginine-NO-cGMP pathway [12], and 5HT_1A_ receptor [11] and mediates the antidepressant-like activity. It is also reported to increase BDNF levels in brain [12]. The different site and mechanism of action of hesperidin with agomelatine or bupropion might have contributed in synergistic effects with combination approach.

Overall, combination treated groups such as hesperidin plus agomelatine, hesperidin plus bupropion, and agomelatine plus bupropion were better in elevating serotonin and dopamine in hippocampi and cerebral cortices and reducing the immobility period. Melatonergic antidepressant agents such as agomelatine have a track record of producing very few side effects as compared to other standard antidepressant drugs like venlafaxine, SSRI's, duloxetine, etc. [35, 40]. It has been reported that agomelatine may cause a dose-related elevated serum transaminases. The combination of hesperidin with agomelatine may help in reducing side/adverse effects of later drug. Though previous reports suggest no impact on locomotion of hesperidin [41], agomelatine [42], and bupropion [43] treatments, the drawback of present study is not considering the evaluation of locomotor activity along with immobility testing. Additional parameters such as BDNF, glutamate, and melatonin should be further investigated in different preclinical and clinical settings as a future endeavour.

## Data Availability

The data used to support the findings of this study is available from the corresponding author upon request.
